# Towards a better understanding of *Lactobacillus rhamnosus *GG - host interactions

**DOI:** 10.1186/1475-2859-13-S1-S7

**Published:** 2014-08-29

**Authors:** Marijke E Segers, Sarah Lebeer

**Affiliations:** 1 University of Antwerp, Department of Bioscience Engineering, Research Group Environmental Ecology and Applied Microbiology, Groenenborgerlaan 171, B-2020 Antwerp, Belgium; 2 KU Leuven, Centre of Microbial and Plant Genetics, Kasteelpark Arenberg 20, box 2460, B- 3001 Leuven, Belgium

**Keywords:** *Lactobacillus rhamnosus *GG, probiotic, bacteria-host interactions, MAMP-PRR, immunomodulation

## Abstract

*Lactobacillus rhamnosus *GG (LGG) is one of the most widely used probiotic strains. Various health effects are well documented including the prevention and treatment of gastro-intestinal infections and diarrhea, and stimulation of immune responses that promote vaccination or even prevent certain allergic symptoms. However, not all intervention studies could show a clinical benefit and even for the same conditions, the results are not univocal. Clearly, the host phenotype governed by age, genetics and environmental factors such as the endogenous microbiota, plays a role in whether individuals are responders or non-responders. However, we believe that a detailed knowledge of the bacterial physiology and the LGG molecules that play a key role in its host-interaction capacity is crucial for a better understanding of its potential health benefits. Molecules that were yet identified as important factors governing host interactions include its adhesive pili or fimbriae, its lipoteichoic acid molecules, its major secreted proteins and its galactose-rich exopolysaccharides, as well as specific DNA motifs. Nevertheless, future studies are needed to correlate specific health effects to these molecular effectors in LGG, and also in other probiotic strains.

## Introduction

*Lactobacillus rhamnosus *GG (LGG), ATCC 53103 was originally isolated from fecal samples of a healthy human adult by Sherwood Gorbach and Barry Goldwin, explaining its typical surname letters GG. It was identified as a potential probiotic strain because of its resistance to acid and bile, good growth characteristics and adhesion capacity to the intestinal epithelial layer [1]. Ever since, it has been one of the most widely studied probiotic strains, used in a variety of commercially available probiotic products. The beneficial effects of this strain have been studied extensively in clinical trials and human intervention studies.

Probiotic bacteria are proposed to benefit human health mainly by three general mechanisms of action [2,3]. Firstly, certain probiotics can clearly exclude or inhibit pathogens, either through direct action or through influence on the commensal microbiota [2,4]. A second mechanism is the capacity of certain probiotic strains to enhance the epithelial barrier function by modulating signaling pathways, such as nuclear factor-κB (NF-kB), Akt and mitogen- activated protein kinase (MAPK)-dependent pathways, which lead to for example the induction of mucus [5], or increased tight junction functioning [6]. Thirdly, most probiotic strains can also modulate host immune responses, exerting strain-specific local and systemic effects [7]. Many of the interactions between probiotic bacteria and intestinal epithelial and immune cells are thought to be mediated by molecular structures, known as microbe- associated molecular patterns (MAMPs), which can be recognized through specific pattern recognition receptors (PRRs) such as Toll-like receptors (TLRs) [8].

Even though many experimental *in vitro *data and experiments in animal models validate these mechanisms for probiotic strains in general and for LGG in specific, most published *in vivo *data in humans pay less attention to mechanisms of action. Nevertheless, we believe that for an optimized and more tailored application of probiotics, it is imperative to understand the mechanisms of interaction with the host in great detail. LGG is an interesting model probiotic strain, because of its wide use, its available genome sequence [9] and the availability of numerous knock-out mutants that allow the study of gene-function relations [10-15]. In this review we aim to give an overview of the recent advances in the molecular knowledge on LGG-host interactions and try to provide a molecular framework for a better understanding of the health effects of LGG.

## Pili-mediated adhesive capacity of LGG

### In vivo and in vitro evidence

**Figure 1:**
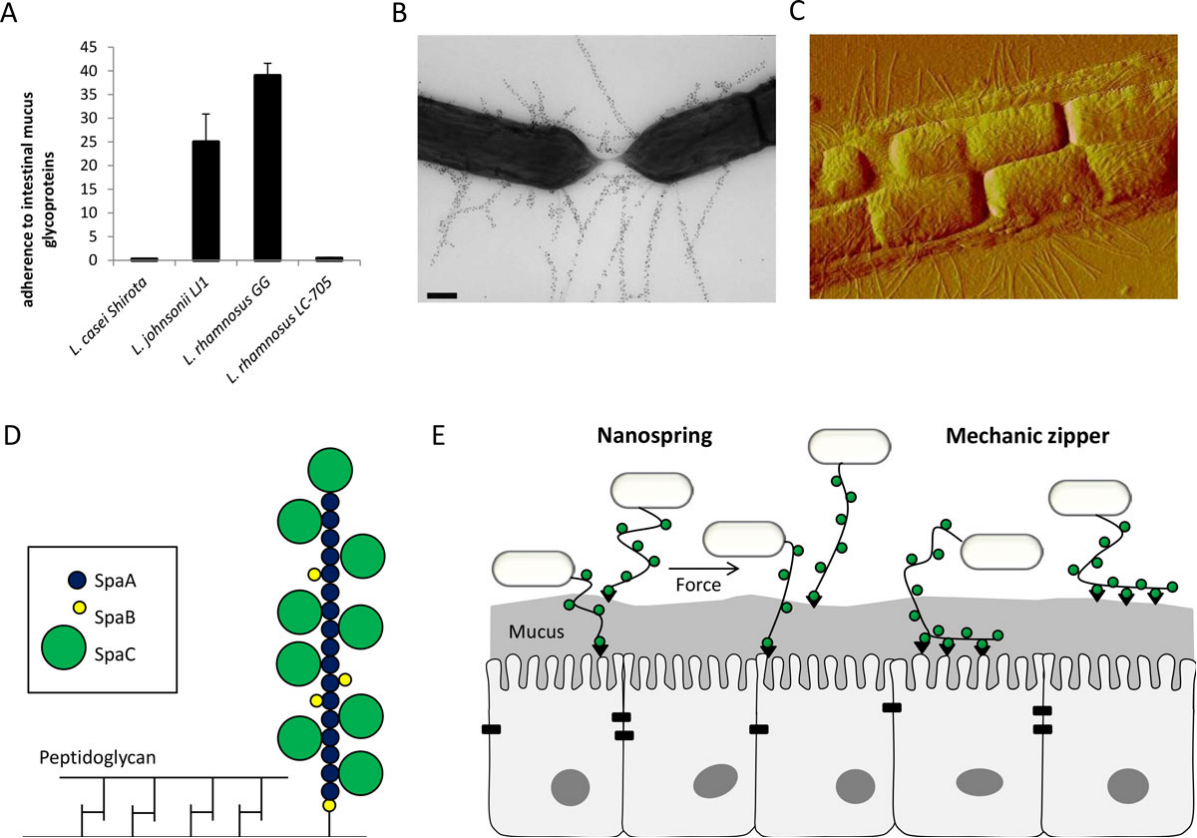
**SpaCBA pili and the molecular mechanisms of adhesion**. LGG is very good mucus adhering Lactobacillus strain compared to other probiotic strains such as L. casei Shirota and L. johnsonii LJ1 and the closely related strain L. rhamnosus Lc705. Radioactively labeled bacteria were allowed to adhere to isolated human intestinal mucus. The adhesion ratio (%) was determined by comparing radioactivity of bacteria added to the radioactivity of bound bacteria after washing (A). Data were published before [16]. Presence of SpaCBA pili LGG cells based on a TEM image of LGG labeled with SpaA antiserum and 10 nm protein A gold particles [26] (B) and on a AFM image of LGG in air [37] (C). The predicted model of the pili shows a pilus backbone formed by the major subunit SpaA, as shown in the schematic figure. The minor subunit SpaC is present on the tip and decorates the pilus over the length at ratio 1:2 with SpaA. The Spa B minor pilin serves as a molecular switch for pilus termination and is bound to the peptidoglycan layer. However, it is suggested that leaky activity of the pilin-specific sortase can include SpaB decorations on the pilus (D). Adapted from [26]. The SpaC pilin is thought to serve as a major adhesin of LGG. It can interact with other SpaC molecules, inducing homophilic adhesion, and with intestinal epithelial cells or their extracellular matrix, in heterophilic adhesion. The exact adhesion sites however remain unkown. Pili can have molecular spring properties which makes them capable to withstand shearing stress. Moreover, the SpaC pilin decorated over the pilus length provide a molecular zipper mechanism that can facilitate a close interaction between the host and the bacteria or bacteria with each other (E). Adapted from [35].

One of the widely studied key features of LGG is its strong adhesive capacity, which has been documented *in vitro *but also *in vivo *in humans. LGG has been shown to be a very good mucus adhering *Lactobacillus *strain compared to related strains such as the dairy strain *L. rhamnosus *Lc705 and other probiotic strains such as *L. johnsonii *LJ1 and *L. casei *Shirota [16] (Figure 1A). In human intervention studies, LGG was also reported to persist longer and in higher concentrations compared to closely related strains such as *L. rhamnosus *LC705 [9]. Orally administered LGG can be recovered from the feces at least one week after administration in adults [9,17]. Of note, the colonization capacity of LGG appears to be significantly better in newborns [18], which is related to reduced colonization resistance exerted by a less established microbiota of infants and which is probably a general feature for many probiotic strains. LGG can adhere to intestinal mucus [9,19] and persist in the descending colon [20]. Colonic biopsies even suggest that LGG colonization continues for longer than indicated by fecal recovery [21]. LGG could also be recovered from the tonsils [22], vagina [23] and oral cavity [24] after probiotic therapy, but it does not seem as efficient as other *Lactobacillus *strains in colonizing these niches. LGG seems to show a preferential tissue tropism for the intestinal mucus layer, although these findings could be biased, as only limited studies have investigated the adherence and colonization at other body sites.

### Pili as key intestinal adhesins

A major breakthrough in our understanding of the excellent adherence capacity of LGG was the discovery of fimbria-like appendages [25], later named as pili [9]. Pili or fimbriae are long and thin proteinaceous protrusions of the cell surface present on specific Gram-positive and Gram-negative bacteria. A genomic comparison with the closely related strain LC705 resulted in identification of the *spaCBA *gene cluster involved in the biosynthesis of the LGG-specific SpaCBA pili, and the confirmation by Western blot and immunogold transmission electron microscopy of structures of ca. 1 µm that are around 5 nm thin (Figure 1B) [9]. Subsequently, we found by mutational analysis of several predicted adhesins that the SpaCBA pili play a key role in adhesion to mucus, the Caco-2 intestinal epithelial cell line and promote biofilm formation [14]. Of note, the LGG genome also encodes another pili gene cluster, *spaDEF*, but these pili do not seem to be expressed, at least not under the tested conditions [9,14,26]. Interestingly, the pili of *Bifidobacterium breve *UCC2003 appear to be only expressed in the murine gastro-intestinal tract and not in laboratory conditions [27], highlighting that condition-dependent expression of the pili cannot be ruled out. However, LGG remains piliated with SpaCBA pili under different stress conditions such as bile salts and low pH [28]. Recently pili were also found in various other lactobacilli and lactococci [29-31]. Remarkably, comparative genome and functional analysis of *L. rhamnosus *species showed that functional SpaCBA pili are significantly more prevalent in human isolates than in dairy isolates. Moreover, the pili are most prevalent in intestinal isolates, while none of the strains that originated from the oral and vaginal cavities were shown to have functional pili [28]. Intriguingly, recent gut metagenomic studies also showed that pili genes form important examples of highly abundant functions that could be identified, even if mainly expressed by some low-abundance microbes such as *Echerichia coli*, further supporting a key role for mucus-binding pili in the intestinal niche [32].

### Structural properties of the SpaCBA pili

The LGG pilus contains three distinct pilin monomers which are covalently linked in a sortase-dependent manner: the major pilin SpaA and the minor pilins SpaB and SpaC (Figure 1.D). Mass spectrometry revealed a SpaA/SpaC/SpaB ratio of 5:2:1 [26]. As observed in other Gram-positive pili, the major pilin SpaA exerts merely a structural function forming the pilus backbone, while the accessory pilins play a functional role [33,34]. The larger minor pilin SpaC, located on the tip of the pilus and covering the pilus length [26], is thought to play a pivotal role in adhesion to mucus. This was shown using whole bacteria with a isogenic *spaCBA *mutation [9], competitive blocking experiments with SpaC antiserum [9], experiments with recombinant SpaC in a mucus binding assay [9] and single molecule atomic force microscopy (AFM) [35]. The smaller minor pilin SpaB is thought to act as a molecular switch responsible for pilus termination and initiation of peptidoglycan binding by the housekeeping sortase [26].

Another interesting feature of pili expression is the presence of numerous insertion sequence (IS) elements flanking the *spaCBA *operon in the LGG genome. It was even suggested that the iso-IS30 element actually enhances pili expression in LGG, while not in *L. casei *strains [30]. It is however also possible that spontaneous removal of the IS element would stop expression of pili in LGG, although Douillard *et al*. [30] showed that LGG could be recovered from various commercial products with a very limited amount of genomic changes (2-3 SNPs) and was still capable of producing pili, indicating its robustness under fermentation conditions. Nevertheless, others showed in a comparative genome analysis of three LGG dairy product isolates that in two of these strains, major stretches of genomic DNA were deleted, including the *spaCBA *operon [36]. Moreover, LGG pili are susceptible to shearing stress. Our AFM study showed that bacterial cells subjected to 8000 × g centrifugal forces are completely devoid of pili [37]. As pili seem to play an important role for the probiotic function of LGG, it is important for industrial production of LGG that detrimental shearing stresses are avoided. The presence of pili should be a key question for future intervention studies with LGG.

Based on single molecule AFM, we could also suggest that the LGG pili have two important nanoscale properties mediating interactions with the host (Figure 1.E). Firstly, pili appear to function as nanosprings capable of withstanding forces such as shearing stress. Secondly, pili can function as a mechanical zipper [35]. As SpaC is distributed not only at the tip, but also over the pilus length [26], it is thought that a zipper-like mechanism can facilitate a close interaction with the host after the first interaction is initiated by a SpaC subunit, enabling other SpaC subunits along the tip, but also other cell surface adhesins on the LGG surface to adhere step by step, resulting in a more stable interaction. For instance, two other LPxTG-like surface adhesins were found in LGG: MabA as modulator of biofilm formation and adhesion [13] and the mucus-binding factor MBF [38].

### Possible immunomodulatory role of the SpaCBA pili

As key adhesive components, the immunomodulatory capacity of the pili is also of high interest, although current data are very limited. In a first study, focusing on the nonpiliated *spaCBA *mutant of LGG, we could show a ca. twofold increased induction of interleukin-8 (IL-8) and other pro-inflammatory markers in intestinal epithelial Caco-2 cells compared to wild-type, while a mutant lacking exopolysaccharide (EPS) and showing an increased exposure of SpaCBA pili resulted in ca. twofold less IL-8 mRNA induction [14]. In a more recent study, *Lactococcus lactis *was genetically engineered to express the LGG SpaCBA pili. These constructs appeared to promote interaction with TLR2 and expression of IL-8 [39]. Moreover, in a comparison between LGG and other commercial probiotic *L. rhamnosus *and *L. casei *strains, it was shown that certain closely related strains lacking pili expression have a reduced interaction with TLR2 compared to LGG [28]. As part of our ongoing research, we are investigating whether the pili have a direct effect as MAMP interacting with PRRs, such as TLR2, like has been shown for the type I pilus of *Streptococcus pneumonia *[40], or whether they mainly act indirect by mediating a close interaction with the host cells and other LGG exposing surface-bound ligands. Recent data suggest that it is not specifically the presence or absence of SpaCBA pili that mediate immune status. Rather, the pili appear to play an indirect modulating role by promoting close interactions with host cells such as epithelial cells, probably by the zipper-like mechanism discussed above, so that other effector molecules can exert their immune modulating activities [14]. It will be very interesting to substantiate this indirect immunomodulatory role in human volunteers, e.g. by intervention studies with spontaneous pili mutants of LGG.

## Lipoteichoic acid (LTA) as key immune effector of LGG

One of the first effector molecules of LGG that were studied are its lipoteichoic acid (LTA) molecules, because LTA is considered as the Gram-positive equivalent of Gram-negative LPS in stimulating strong immune responses. However, the bio-active concentration of LTA is typically in de micromolar range, while LPS is active in nanomolar concentrations [41]. Moreover, LPS and LTA interact with different PRRs. The specific PRRs for LTA were shown to consist of TLR2 in a heterodimer with TLR6 and co-receptors CD14 and CD36 [42]. The structure of LTA of LGG was shown by NMR to consist of a glycerolphosphate backbone with D-alanyl esters (ca. 70%) as unique detectable substituents and an average chain length between n = 30 and n = 76. The glycolipid moiety contains 2 fatty acid chains with an average length of C14, with one double bound per fatty acid [12,43]. Analysis of structure-activity relations showed the importance of the lipid chains of LTA in LGG in interaction with TLR2-6 and the induction of NF-κB signaling [43].

To investigate the *in situ *role of LTA in live bacteria, a mutant of LGG that showed a modified LTA structure lacking D-alanine residues and an altered glycolipid anchor, was created by mutating the *dltD *gene. This gene encodes a membrane protein that facilitates the ligation of the D-alanyl carrier protein with D-alanine [12]. This mutant showed a strongly reduced interaction with TLR2-6 and a lower induction of IL-8 mRNA in the Caco-2 cell line compared to wild type, further substantiating a key role for LGG LTA in promoting more pro- inflammatory responses [43]. Furthermore, the *dltD *mutant significantly improved DSS- induced colitis in treated mice compared to buffer control, while the wild type strain showed actually detrimental effects in that model [44]. Interestingly, similar effects were observed with LTA mutants in other lactobacilli [45]. Complete removal of LTA in *Lactobacillus acidophilus *NCFM [46] or a shift from mainly D-alanine to glucosyl substitutions in *Lactobacillus plantarum *NCIMB8826 [47] also resulted in strains capable of more efficiently alleviating inflammation. This shows that LTA is not an LGG-specific effector molecule, but that it is an important molecule in understanding *Lactobacillus*-host interactions and is a crucial factor to take into account when investigating anti-inflammatory effects.

## Secreted proteins as probiotic effectors

In 2002, Yan and Polk showed that LGG promotes the survival of intestinal epithelial cells (IECs) by preventing cytokine-induced apoptosis through blocking of p38 MAP kinase. They found that the survival-promoting effect was also present in other probiotic strains such as *L. acidophilus *ATCC393 and *L. casei *ATCC4356, but the strongest in LGG [48]. The supernatant of LGG was shown to prevent apoptosis in IECs [49] and induce heat shock proteins [50]. Consequently, two proteins from the LGG supernatans were found to cause the antiapoptic effect [49]. These proteins p75 (~75 kDa) and p40 (~40 kDa) were later renamed as Major Secreted Protein Msp1 and Msp2 respectively, because of the discrepancy in molecular weight [15]. Each of the purified proteins was shown to activate the Akt signaling peptide, inhibit cytokine-induced IEC apoptosis and reduce tumor necrosis factor (TNF)- induced epithelial damage. The proteins also promoted cell growth in human and mouse colon IECs and cultured mouse colon explants [51]. Moreover, they were shown to protect the intestinal epithelial barrier function from hydrogen peroxide-induced damage through blocking of MAP kinases [52].

A subsequent *in vivo *mice study proved the efficacy of recombinant Msp2/p40 delivered in a pectin/zein hydrogel bead for the prevention and treatment of DSS- induced intestinal injury and acute colitis and for ameliorating colon epithelial cell apoptosis and chronic inflammation in oxazolone-induced colitis [53]. Moreover, Msp2/p40 was shown to cause phosphorylation of the epidermal growth factor receptor (EGF-R) leading to activation of Akt. The importance of EGF-R in the mechanism was confirmed in the *in vivo *tests [53]. Nevertheless, the exact PRR could not be identified. The activation of EGF-R is thought to be indirect as Msp2/p40 was shown to stimulate the catalytic activity of the metalloproteinase ADAM-17, which subsequently releases heparin-bound EGF in IECs, activating EGF-R [54].

As mentioned before, Msp1/p75 and Msp2/p40 are not unique for LGG. For instance, the homologous proteins of *L. casei *BL23 can also cause EGF-R phosphorylation [55]. However, there exists some heterogeneity between the homologues. Analysis of Msp1/p75 and Msp2/p40 gene sequence showed homology with cell wall hydrolases in LGG [51], but also in *L. casei *[55]. Msp1 was characterized in LGG as a D-glutamyl-L-lysyl endopeptidase with an important role in daughter cell separation [15]. Intriguingly, we could also show that the Msp1 protein is glycosylated in LGG with Con-A reactive residues [56], which could explain the apparent discrepancy previously seen between the predicted molecular weight and the molecular weight shown on Western blot [51]. The serine-rich glycosylation site was not found in homologues of Msp1 in several *L. casei *strains, which suggest a species-specific glycosylation. The glycosylation does not impede enzyme activity and was suggested not to interfere with activation of Akt, although a the glycan chain appears to have a modulating role as shield. The glycosylation seems to play a role in increasing protein stability and protein binding to the cell wall [56]. Interestingly, the ConA reactive S-layer protein SlpA of *L. acidophilus *is suggested to be recognized by DC-SIGN [57], although this remains to be further substantiated. The Msp2 protein, on the other hand, appears not to be glycosylated. The exact function of LGG Msp2 remains unclear since its hydrolytic peptidoglycan (PG) degrading activity is limited and an *msp2 *knock-out mutant could not be constructed, possibly because of its essential role in LGG. Immunofluorescence analysis suggests a possible role in early stage cell septum formation [15].

Because of their action as PG hydrolases, it also remains to be studied whether Msp1 and Msp2 could have immunomodulatory functions by release of PG fragments. Intriguingly, a recent study showed that PG might be a central mediator for the beneficial effect of certain probiotic lactobacilli, such as *Lactobacillus salivarius *Ls33 in inflammatory bowel disease with NOD-2 as a key receptor [58]. Although the PG of most lactobacilli share the same basic structure, the PG molecules of this *Lactobacillus *strain was shown to contain an additional muropeptide, MurNAc-l-Ala-γ-d-isoGln-l-Lys, which was suggested to be the NOD2 ligand displaying anti-inflammatory properties [59]. The PG structure of LGG was previously determined when analyzing the PG hydrolyzing activity of Msp1/p75 [15]. It is at present not known whether LGG PG contains such strong NOD2 ligands and how this NOD2 interaction could be influenced by its collection of PG hydrolases.

## Exopolysaccharides (EPS) as modulating adaptation factors

An extracellular polysaccharide (EPS) layer is commonly found in lactobacilli. Of interest, EPS molecules show a large structural diversity [60], so that they are clearly strain-specific molecules. The LGG cell surface appears to contain two major types of polysaccharides: long galactose-rich polysaccharides and shorter glucose/mannose-rich polysaccharides [61]. We could yet identify the operon responsible for galactose-rich EPS synthesis [62]. A knock-out mutant of the *welE *gene, encoding the priming glycosyltransferase, is completely devoid of the long galactose-rich EPS but shows a higher concentration of short glucose-rich EPS [62]. This mutant shows an increased adhesion to Caco-2 IECs, linked to increased exposure of the SpaCBA pili [14,62]. In addition, this type of galactose-rich EPS appears to be an important adaptation factor for LGG as the *welE *mutant shows a reduced *in vivo *survival in the murine gastrointestinal tract (GIT) [63]. More specifically, the long galactose-rich EPS were shown to protect the cell against complement-mediated lysis and cathelicidins, specific cationic antimicrobial peptides [63]. It thus seems important that this type of EPS production is balanced between optimal protection and optimal adhesion.

The role of EPS in the interaction between LGG and the host remains largely unclear. Results obtained in our lab indicate that isolated galactose-rich EPS are not principal inducers of cytokines in the Caco-2 intestinal epithelial cell line [14]. On the other hand, isolated EPS from LGG appears to counteract the cytotoxicity of *Bacillus cereus *on Caco-2 cells and of streptolysin-O on rabbit erythrocytes [64]. However, as low concentrations of the major secreted proteins have an anti-apoptotic effect in IECs [51], it remains to be ruled out that protein contaminants in the purified EPS are not interfering with the results. In addition, the role of the other glucose-rich type of EPS from LGG remains to be substantiated. A recent network based *in silico *analysis of the glycosyltranferase genes of LGG could identify the putative gene cluster involved in their biosynthesis [65], which opens perspectives for functional analyses with mutants.

Apart from EPS, other genes and molecules play a role as factors that promote the adaptation of LGG to the human host and gastro-intestinal tract in particular. For instance, an elegant study combining transcriptomics and proteomics in LGG identified putative adaptation factors involved in the bile stress response of LGG. Among the identified functions were general stress responses as well as cell envelope-related functions, including pathways affecting fatty acid composition, cell surface charge, and thickness of the EPS layer [66]. Our recent recombinase-based *in vivo *expression technology (R-IVET) experiment, similar as in *L. plantarum *WCFS1 [67], also indicated a remarkable metabolic flexibility of LGG in the murine gastro-intestinal tract (Sarah Lebeer *et al*., in preparation).

## Secreted antimicrobials produced by LGG

Several *in vitro *studies have shown the efficacy of LGG against the viability, adherence or infection of GIT pathogens. Indeed, LGG was shown to reduce the viability of *Salmonella enterica *subsp. e*nterica *serovar Typhimurium [68-72], *Shigella sonei *[73], and *Pseudomonas, Staphylococcus *and *Streptococcus *strains [70]* in vitro. In vivo *mice experiments confirmed that LGG pretreatment reduces *S*. Typhimurium infection parameters [74]. However, there is some controversy concerning the results obtained with *S*. Typhimurium, as not all studies could detect a reduction in viability [74,75] or reduced adherence to human intestinal mucus [75,76]. Unfortunately, to our knowledge no human trials have been carried out focusing on *Salmonella *specifically.

There have been a number of studies trying to identify the antibacterial compounds, mostly focusing on *S*. Typhimurium. LGG grown in de Man-Rogosa-Sharpe (MRS), a medium recommended for lactobacilli, was shown to inhibit growth of *S*. Typhimurium SL1344 in a pH-dependent manner [76]. Interestingly, lactic acid was identified as the main antimicrobial compound in different conditions [69,71,72], which is clearly not a specific factor for LGG. As lactic acid permeabilizes the Gram-negative outer membrane, it might facilitate antibacterial action of other compounds, such as organic acids or bacteriocins [77]. These compounds might indeed participate as a recent study showed that the antimicrobial effect appears not to be dependent on lactic acid concentration alone [68]. Seven heat-stable peptides with antibacterial activity against enteroaggregative *E. coli *strain EAEC 042, *Salmonella *Typhi, and *Staphylococcus aureus *were identified in LGG culture medium [78]. Unfortunately, these peptides have not yet been identified, to the best of our knowledge. Of note, the genome sequence of LGG encodes several bacteriocin-related genes [9]. Despite several attempts, we and others were not yet able to shown bacteriocin production under laboratory conditions and coculture with possible inducing strains, although a bacteriocin locus was found to be induced in the murine gastro-intestinal tract after R-IVET (Sarah Lebeer *et al*., in preparation).

Bacterial cell-cell communication through quorum sensing (QS) might also interfere with pathogen infection as strains present in the gut microbiota are thought to communicate to coordinate adaptive processes such as competition and cooperation for nutrients and adhesion sites [2]. LGG was reported to produce autoinducer-2 (AI-2), which is suggested to be an important interspecies QS molecules, produced by both Gram-positive and Gram-negative bacteria [79]. However, the role of QS in pathogen exclusion is difficult to investigate since the AI-2 synthase LuxS also interferes with the cell metabolism. Indeed, a *luxS *knock-out mutant of LGG was shown to have numerous pleiotropic effects, which could not be complemented by exogenous addition of synthetic AI-2 molecules [10]. It remains to be investigated whether AI-2 or other QS systems play a role as a probiotic mechanism for LGG. For instance, McCormick and colleagues could nicely show that cyclic dipeptides of strain *Lactobacillus reuteri *RC-14 quench *agr*-mediated expression of toxic shock syndrome toxin- 1 in staphylococci [80], highlighting that QS could play a role in antipathogenic mechanisms of probiotics.

## Unmethylated CpG-rich DNA motifs as intracellular MAMPs

Other important bacterial MAMPs are derived from bacterial DNA and become only available after cell lysis. Bacterial DNA can be distinguished from eukaryotic DNA in frequency of unmethylated cytosine-guanine dinucleotides (CpG) motifs. These CpG motifs are relatively widespread in viral and bacterial DNA, but are not common in mammalian DNA. CpG motifs and synthetic unmethylated CpG oligonucleotide mimics (ODN) are generally recognized by TLR9 and can induce a strong T-helper-1 (T^H^-1) like inflammatory response [81]. Targeting TLR9 with CpG or ODN has been a strategy for a number of clinical trials studying the effect on cancer treatment, allergy and infection diseases, reviewed in [82]. It is important to note that TLR9 function in the intestinal epithelial layer is thought to be polarized as IECs respond differently to apical or basolateral exposure to CpG. As basolateral TLR9 activation signals activation of the NF-κB pathway, apical TLR9 stimulation seems to prevent NF-κB activation. This mechanism is thought to play an important role in epithelial homeostasis [83].

A bioinformatic analysis of the frequency of CpG motifs in the genomes of gut commensals demonstrated a correlation with genomic GC content [84]. Indeed, *in vitro *treatment of polarized IEC layers showed that DNA form different probiotic strains have differential effects on NF-κB activation [85] and *in vivo *studies using a mouse model showed differential effects on immune proliferation activity [86]. The genome of LGG, but also of other lactobacilli such as *L. plantarum *WCFS1, appears to have a higher frequency of the optimal motif for interaction with TLR9, i.e. GTCGTT, than could be expected by their genomic GC content [84]. Moreover, a potent ODN, TTTCGTTT named ID35, was identified in the LGG genome [86]. The effect of chromosomal DNA of LGG was also tested in polarized IECs, where it was shown to diminish TNF-induced NF-κB activation and reduction of trans- epithelial resistance thus protecting the epithelial layer [87]. The chromosomal DNA of LGG and derived ODNs were also shown to be strong *in vivo *inducers of murine B cell proliferation and were able to stimulate T_H_1 immunity in murine splenocyte cells [86]. ID35 isolated form LGG genome even seems to be beneficial in allergy prevention in an ovalbumin-sensitized mouse model, by inducing the T_H_1-response and suppressing ovalbumine-specific IgE production [88]. Moreover, a study using peripheral blood mononuclear cells (PBMCs) from allergic patients showed that LGG as well as its genomic DNA can modulate the T_H_1/T_H_2 response to specific allergens dose-specifically. More than 50% of the effect of LGG could be explained by the effect of the genomic DNA, as stated by the authors [89].

## Profiling of host responses against LGG

**Figure 2:**
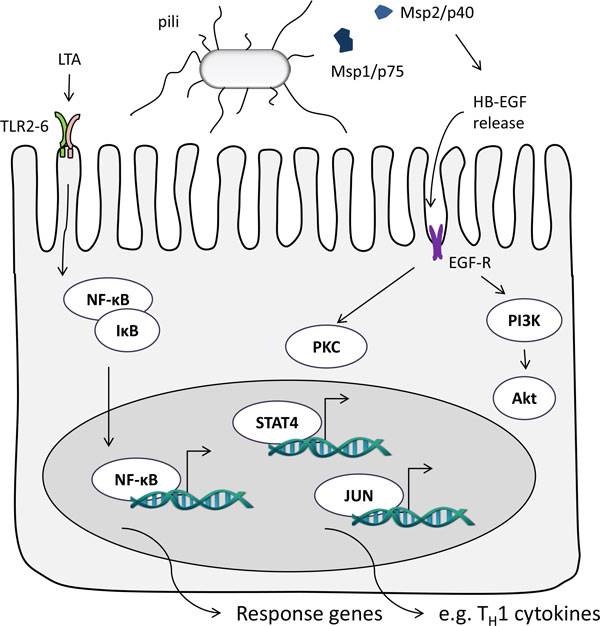
**Molecular interactions of LGG with intestinal epithelial cells**. LTA as a MAMP interacts with TLR2-6, activating NF-κ signaling [43]. Secreted protein Msp2/p40 induces release of HB-EGF that causes phosphorylation of EGF-R, activating downstream protein kinase C (PKC) and phosphoinositide 3-kinase (PI3K) -Akt signaling [51,53,54]. A recent human duodenal transcriptome study indicates that JUN and STAT4 transcription factors play a central role in downstream signaling after consumption of LGG, leading to mainly T**_H_**1 cytokine production and activating pathways involved in cellular growth and proliferation, wound healing, angiogenesis, interferon-mediated responses, calcium signaling and ion homeostasis [92]. Adapted from [96]

The above mentioned LGG molecules and their corresponding mutants are studied one-by- one but it should be highlighted that *in situ *the host interaction towards LGG will be an integrated sum of different interactions. A combination of all MAMP-PRR interactions decides how the immune system is triggered, while also various metabolites such as lactic acid can be envisaged to modulate host responses. Therefore, molecular profiling of the host responses upon LGG application can reveal important novel insights (Figure 2), especially if time course studies are included. Until now, these host responses towards LGG have been mainly characterized by transcriptomics methods, but also other approaches such as proteomics and metabolomics show great potential, especially if methods are integrated and combined with network biology approaches [90]. For instance, a gene expression analysis of the small bowel mucosa from patients treated with LGG compared with placebo treatment, showed that LGG affected genes involved in immune response and inflammation, apoptosis, cell-cell signaling, cell growth and cell differentiation, cell adhesion and signaling. It should be noted that these analyses were done at a rather late time point, i.e. in biopsy samples of patients consuming LGG during one month (1.2 × 10^10 ^colony forming units, CFU, daily) [91]. A more recent *in vivo *transcriptome analysis compared the mucosal responses towards LGG (1.68 × 10^10^) with two other commercially available lactobacilli (i.e. *L. acidophilus *Lafti-L10, *L. casei *CRL-431) in a placebo-controlled randomized double-blind cross-over design in which the volunteers consumed all three probiotic preparations and a placebo control in a randomized order with each time a 2-week wash-out period. Interestingly, even after only 6 h, the mucosal response to LGG was also mainly characterized by the induction of T_H_1 development via the IFN-STAT4 (signal transducer and activator of transcription 4) axis and affected pathways include cellular growth and proliferation pathways, wound healing, angiogenesis, interferon mediated responses, calcium signaling and ion homeostasis [92]. These pathways contain signatures of the previously documented activity of Msp1/p75 and Msp2/p40 to promote cell proliferation and epithelial integrity [51,53,54], but clearly other factors play a role. The same method was also used to investigate whether humans respond differently to different growth stages of *L. plantarum *WCFS1 [93]. They indeed observed clear differences in the transcriptional response to exponentially growing or stationary phase bacteria, and between viable and heat-killed stationary bacteria. It will be very interesting to use the same analyses to investigate the transcriptional responses towards LGG wild type and spontaneous non-GMO food-grade mutants, such as spontaneous pili mutants, to explore their relative contribution to the human host response. Alternatively, experiments with dedicated isogenic mutants such as of Msp1/p75 [15] could be designed for analyses in animal models, since Lin *et al*. [94] have also nicely shown by transcriptomics that LGG also upregulates cytoprotective gene expression and MAPK-related expression in the developing murine intestine. In addition, *ex vivo *and *in vitro *models such as the porcine small intestinal epithelial cell line (IPEC-J2) appear to be good models for the study of innate immune responses to probiotics [95].

Nevertheless, and probably most importantly, the abovementioned duodenal transcriptome studies of Kleerebezem and coworkers showed a remarkable large distance of the transcriptome profiles between the human participants. As all participants were healthy, the large inter-person variation indicates that mucosal tissues have multiple mucosal solutions to accomplish healthy homeostasis, which is suggested to be a molecular "bandwidth of human health" [96]. Clearly, this complicates the selection of the most appropriate biomarkers to monitor probiotic intervention in human study subjects and the possibility for stratification of responders and non-responders.

## Clinical benefits of LGG

In the next paragraphs, we tried to summarize some well documented clinical benefits and explain some of the findings with the molecular framework we provided above, although this association should be taken with caution.

### Promotion of gastro-intestinal health in children and adults

As mentioned before, LGG has been shown to colonize the gut of newborns significantly better than adults [18]. Interestingly, prenatal supplementation with LGG (1.8×10^10 ^CFU in capsules, daily from 36^th ^week of gestation) has been reported to change the composition of the neonates microbiota, promoting a beneficial profile dominated by bifidobacteria [97,98], although the overall microbial diversity did not seem to have changed [99]. Others showed that postnatal application of LGG (10^9 ^CFU daily, lyophilized powder mixed with breast milk) appears to affect neonatal intestinal colonization patterns causing a higher species diversity compared to placebo [100], although the analysis was not done at detailed level. How exactly LGG could promote the colonization of bifidobacteria *in vivo *remains to be further explored.

Given its excellent intestinal mucus adherence capacities, LGG has also been often selected as candidate probiotic for the prevention and treatment of gastro-intestinal infections and diarrhea, although the efficacy is not uniformly proven. Three subsequent meta-analysis studies have discussed the use of LGG for the treatment of acute diarrhea in children [101-103]. Overall, the current data suggest that LGG can reduce the duration of diarrhea with 1.05 days, particularly in children from geographical Europe, treated with a high dose of LGG (≥10^10 ^CFU/day). LGG (10^9 ^CFU daily in fermented milk product) was also shown to reduce the risk of acquiring nosocomial gastrointestinal infections when administered daily in hospitalized children [104]. Undernourished Peruvian children showed a lower incidence of diarrhea when treated with LGG (>10^10 ^CFU daily, lyophilized powder mixed with liquid cherry gelatin). Effects were the largest in non-breastfed children [105]. However, long-term consumption of milk containing LGG (10^8 ^CFU daily) in children attending day care centers in Finland could not show an effect on the incidence of gastro-intestinal symptoms [106]. It is important to note that the unsuccessful trial tested a 100-fold lower daily concentration, although other factors such as probiotic formulation and study subject heterogeneity could of course also play a role. A recent meta-analysis concluded that LGG treatment can also reduce pain frequency and intensity in children with abdominal pain-related disorders, particularly among irritable bowel syndrome (IBS) patients. However, it is important to mention that the clinical effects were significant but moderate [107], which is not unexpected if you consider the large subject heterogeneity in IBS patients.

In other conditions, LGG effects were better stratified. For instance, oral supplementation with LGG (6 × 10^9 ^CFU with human milk) has been shown to prevent enteric colonization by *Candida *species in preterm neonates in a randomized study [108], although the underlying mechanisms need to be explored. Two pilot studies also showed promising results for LGG treatment (respectively 10^10 ^CFU in skim milk and 1.2 × 10^9 ^CFU in lyophilized powder daily) of recurrent *Clostridium difficile *induced colitis in children [109,110], but this should be repeated in larger trials. Furthermore, application of a commercial yoghurt with LGG to renal patients during 8 weeks has been shown to succeed in clearing vancomycin-resistant enterococci in all patients in an double-blind, randomized, placebo-controlled trial [111]. A larger single-blind, randomized, placebo-controlled trial focused on children (3 × 10^9 ^CFU daily, dissolved in water or milk) and could show a significant difference between the treated and the control groups only after three weeks [112]. The mechanism of clearing of this vancomycin-resistant enterococci remains to be explored, but the SpaCBA pili of LGG share some sequence identity (30-40%) with the pili of *Enterococcus faecalis *and *Enterococcus faecium *[9]. Clearly, experiments with non-GMO pili deficient variants of LGG would be highly interesting to study their role in the gastro-intestinal and pathogen exclusion effects of LGG.

### Possible effects at other body niches

Although many intervention studies with LGG are targeting the GIT, it is also interesting to investigate extra-intestinal effects of LGG. For instance, LGG has been shown to reduce oral counts of *Streptococcus mutans*, a bacterium correlated with caries formation, respectively in yogurt, milk and lozenges [113-115]. Especially long-term consumption of LGG containing milk (5-10 × 10^5 ^CFU) appears to be able to reduce caries development in children [114]. Of note, there was no effect of short-term consumption of LGG (4×10^8 ^CFU daily) on the acidogenicity of plaque nor on caries formation in adults, although it should be noted that LGG was administered in a tablet, which might not be the best formulation [116]. Importantly, LGG appears not to ferment sucrose to a significant level [9], indicating that it is itself not cariogenic, a property which is sometimes attributed to lactobacilli due to lactic acid production.

Others have investigated the effect of LGG consumption on respiratory health. For instance, Hojsak and colleagues [104] showed that fermented milk containing LGG was efficient in reducing the risk on respiratory tract infections (RTIs) that lasted longer than three days in hospitalized children. Also, preterm infants treated daily with 10^9 ^CFU LGG in capsules starting within one week after birth, appear to have significantly lower incidence of RTIs and rhinovirus-induced episodes in the first 2 months [117]. Furthermore, capsulated LGG (10^9 ^CFU) was shown to protect hospitalized patients against ventilator-associated pneumonia, mainly when caused by Gram-negative pathogens like *Pseudomonas aeruginosa *[118]. Moreover, in cystic fibrosis patients colonized with *P. aeruginosa*, long-term LGG treatment (6×10^9 ^CFU daily, in oral rehydration solution) significantly decreased the incidence of pulmonary exacerbations and increased body weight [119]. Unfortunately, this study did not evaluate *P. aeruginosa *colonization status after LGG treatment. Clearly this area requires further research, because probably a combination of LGG's antipathogenic and immune modulating capacities determines its potential in RTIs.

### Immunomodulatory applications of LGG

#### Allergic diseases

The potential immunomodulatory effects of LGG that have yet received most attention include its widely discussed effects against allergic disease. In a study published in The Lancet, Kalliomäki and colleagues [120-122] showed that the combination of prenatal maternal (2-4 weeks) and postnatal pediatric (6 months) LGG treatment (10^10 ^CFU daily, capsules or in water) in families with a history of atopic disease, significantly lowered the risk of eczema at the age of 2, 4 and 7. However, allergic rhinitis and asthma tended to be more common in the LGG treated group and no significant differences were found in incidence of cow milk allergy. Moreover, Kopp and colleagues [123] could not repeat the beneficial results against eczema using a similar protocol and concentration. The reason for these different outcomes is unknown, however it is thought that the different genetic background of the tested populations (Finnish versus German) might play a role. Also, the German trial had more infants with older siblings, which could be a potential cofounder [120,123]. In addition, it seems that different probiotic products have been used for these studies, so that also differences in probiotic formulation, and for instance pili presence, cannot be ruled out.

Atopic dermatitis in children could not be treated by LGG in three independent trials, using a daily concentration of 5×10^9 ^CFU/100 ml formula [124], 5×10^9 ^CFU in milk [125] or 10^10 ^CFU in milk [126]. However, in these trails there was a consistent but not significant effect of LGG in the IgE-sensitized subgroup. Two other trials also reported that treatment (5×10^9 ^CFU daily in milk) was efficient in IgE-sensitized infants, but not in non-IgE-sensitized infants [127,128]. This is probably a good example that patient stratification is important to identify potential responders, but more research is necessary to determine the effect of LGG in IgE-sensitized infants.

Related to food allergy, it was reported that administration of capsulated LGG (5×10^9 ^CFU) in infants with cow's milk allergy augments IFN-γ production in stimulated PBMCs, thus possibly providing beneficial T_H_1 immunomodulatory signals [128]. Indeed, infants acquire more oral tolerance when hydrolyzed casein formula was administered in combination with LGG (10^7 ^CFU/ 100 mL) than with the formula alone [129]. In milk-hypersensitive adults, LGG (2.6×10^8 ^CFU daily in milk) has been shown to reduce the immunoinflammatory response by reducing the expression of specific receptors such as the complement receptors CR1 and CR3 [130].

#### LGG as a vaccine adjuvant

Another perhaps more elegant way to investigate the immunomodulatory effects of LGG is by studying its capacity to ameliorate humoral responses to vaccines when applied as an adjuvant. One study showed that the immunogenicity of an oral rotavirus vaccine was significantly ameliorated when mixed with 5×10^10 ^CFU of LGG [131]. LGG in milk (10^10 ^CFU daily, 1 week before vaccination, 4 weeks after) was also shown to increase the poliovirus neutralizing antibody titer with a fourfold increase in poliovirus-specific IgA in adults receiving an oral vaccine against polio 1, 2 and 3 [132]. Moreover, LGG treatment (10^10 ^CFU in a capsule, daily, 28 days starting at vaccination) increased protection rates after an oral life attenuated influenza vaccine. The effect was viral strain-dependent as antibody titers against H1N1 and B strains were low for placebo and LGG-treated groups. For the H3N2 strain, LGG increased protection significantly [133]. However, there was no influence on the effect of an oral *S*. Typhi Ty21a oral vaccine (4×10^10 ^CFU daily, 7 days) [134]. In addition, a recent study even showed that maternal supplementation with LGG (1.8 × 10^10 ^CFU daily) from 36 weeks gestation until delivery reduces vaccine-specific immune responses for tetanus, *Haemophilus influenzae *type b (Hib) and pneumococcal conjugate (PCV7) vaccines in infants at high risk of developing allergic disease [135], indicating that the timing of administration is important if one desires an adjuvant effect and that LGG might not always be the best choice for these purposes. Moreover, van Baarlen *et al*. [92,93] showed other lactobacilli such as *L. plantarum *WCFS1 show a more clear modulating of the NF-κB pathway.

## Conclusions

**Figure 3:**
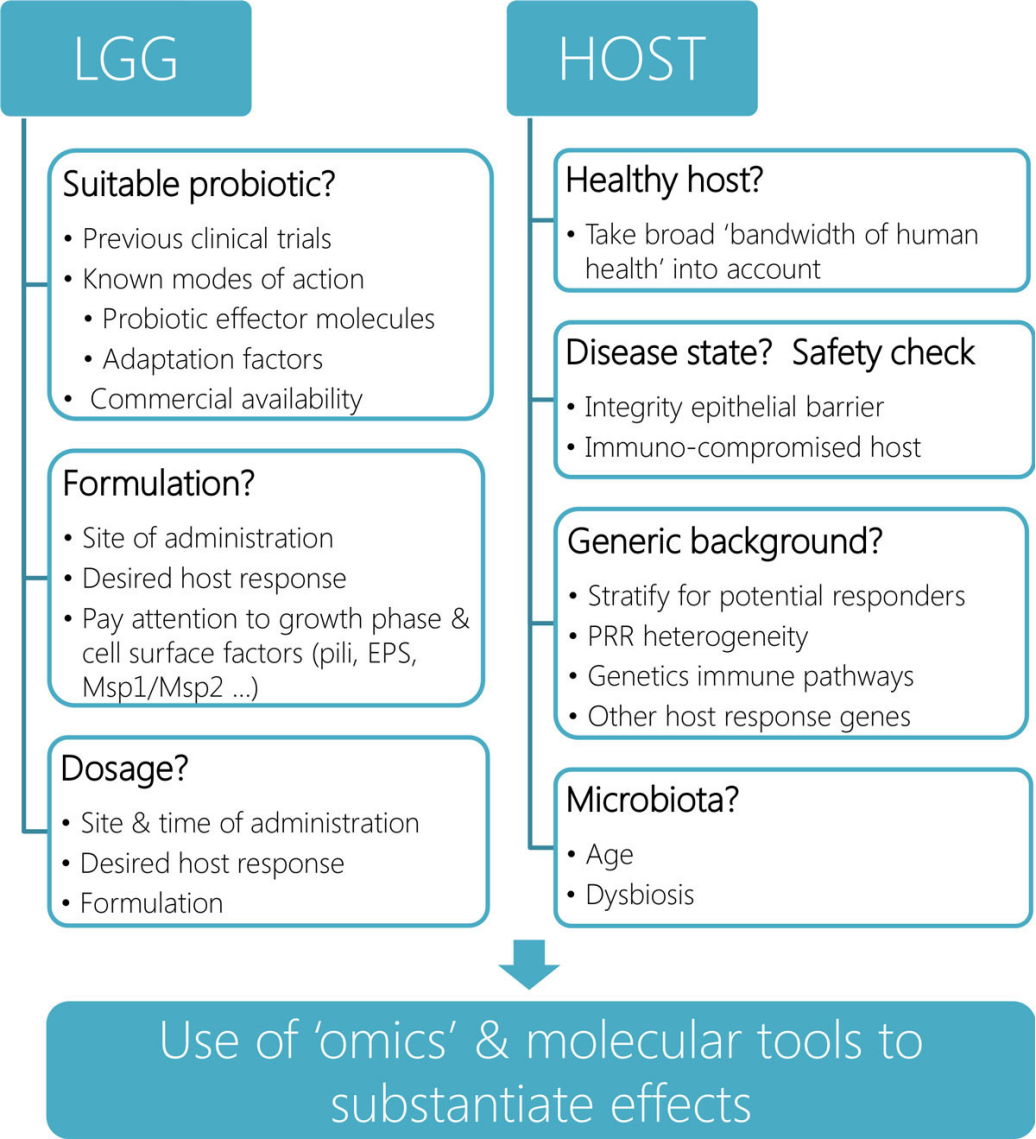
**Pipeline for the design of intervention trials with LGG and related probiotics**. In this schema, we have made an overview of different steps that should ideally been taking when designing novel intervention studies with LGG or related probiotics, taking current information into account. For more information, the reader is referred to the main text of this manuscript.

Is LGG a better probiotic strain than other probiotics on the market? This question is difficult to answer, since the answer largely depends on the host response that is aimed for by the application. As mentioned before, the host response is dependent on the combination of several bacterial effectors, including MAMPs interacting with PRRs. Even though these effectors might not be unique for LGG, it seems that the sum of effectors in LGG is often beneficial for the host, while strains with similar MAMPS might show different results. Small variations in structure (pili, LTA, EPS, etc.), expression level or ratio can also have a large effect on the host response. However, it is also apparent that not all reported health effects of LGG are univocal. Successful administration appears to depend on the applied dose, growth phase, formulation, time of administration, duration of treatment, age and genetic background of study subjects, among other variables (Figure 3). Nevertheless, one of the clear advantages of LGG is that this probiotic is well characterized and so widely used that it has a very good safety track record. LGG has been consumed in over 40 countries worldwide and is especially popular in Finland with a yearly per capita consumption rate of 6L in 2000 [136]. To support the safety of LGG, it was shown that despite increasing LGG consumption in Finland and Sweden respectively, the rate of *Lactobacillus *bacteremia remained constant [137,138]. Moreover, the use of LGG in a wide variety of clinical trials without serious adverse events confirmed its safety. LGG has been administered to, among others, low birth weight infants [139,140], pregnant women [122,123,141], HIV- infected patients [138] and patients with a mechanical ventilator [118].

Nevertheless, case reports show that probiotic therapy is to be discouraged in certain groups of patients. In a review concerning the safety of probiotics, it was concluded that adverse effects of probiotics were correlated with (i) impaired intestinal barrier function, (ii) immune compromised state and (iii) central venous catheter [142]. Indeed, cases specifically reported for LGG show that these risk factors might play a role. For instance, a number of infants treated with LGG for short gut syndrome associated with intestinal friability seemed to manifest sepsis with LGG-like bacteria [143,144] and an ulcerative colitis patient was diagnosed with LGG bacteremia [145], although these identifications were not done at a detailed genomic level. Nevertheless, in patients with a seriously compromised integrity of the barrier function of the intestine, the administration of specific LGG molecules is probably a better strategy than living LGG cells, because of the risk for translocation of the bacteria from the intestines into the blood.

Taken together, the various clinical trials that have yet been published with LGG, notwithstanding their outcome, will help the design of novel trials, while also the recent molecular data on the genes and molecules of LGG that could be important for its probiotic function will lead to better clinical trials and better substantiation of potential modes of actions. This will go hand in hand with the ongoing developments of omics technologies (metagenomics, transcriptomics, metabolomics, detailed sequencing of spontaneous mutants of LGG) to monitor the impact of LGG application and to go towards a better understanding of responders and non-responders.

## List of abbreviations used

AFM: Atomic Force Microscopy; CFU: Colony forming units; CpG: Cytosine-guanine dinucleotide; DC: Dendritic cell; EGF-R: Epidermal growth factor receptor; EPS: Exopolysaccharide; GIT: Gastrointestinal tract; IEC: Intestinal epithelial cell; IS: Insertion sequence; LTA: Lipoteichoic acid; LGG: *Lactobacillus rhamnosus *GG; MAMP: Microbe-associated molecular pattern; PBMC: Peripheral Blood Mononuclear Cells; PG: Peptidoglycan; PRR: Pattern recognition receptor; QS: Quorum sensing; VRE: vancomycin-resistant enterococci.

## Competing interests

The authors declare that they have no competing interests.
